# Adjuvant Effect of Titanium Brushes in Peri-Implant Surgical Treatment: A Systematic Review

**DOI:** 10.3390/dj9080084

**Published:** 2021-07-30

**Authors:** Francisco Javier González, Estefanía Requena, Lucía Miralles, José Luis Sanz, Javier Barberá, José Juan Enciso, Carolina Larrazábal, María Dolores Gómez

**Affiliations:** 1 Deparment of Dentistry, Faculty of Medicine and Health Sciences, Universidad Católica de Valencia San Vicente Martir, 46001 Valencia, Spain; est.requena@gmail.com (E.R.); lucia.miralles@ucv.es (L.M.); javier.barbera@ucv.es (J.B.); joseencisoripoll@hotmail.com (J.J.E.); carolina.larrazabal@ucv.es (C.L.); mariadolores.gomez@ucv.es (M.D.G.); 2 Department of Stomatology, Faculty of Medicine and Dentistry, Universitat de València, 46010 Valencia, Spain; Jsanzalex96@gmail.com

**Keywords:** peri-implantitis, peri-implant surgery, decontamination, implant surface, titanium brush, systematic review

## Abstract

Background: the prognosis of peri-implant surgery can be affected by poor decontamination of the implant surface, which could be improved with the use of titanium brushes. The objectives of this systematic review were to evaluate the effectiveness of titanium brushes in the decontamination of the implant surface in terms of plaque index, probing depth, bleeding on probing and bone loss/gain; as well as its effectiveness according to the type of peri-implant bone defect. Methods: an electronic search was carried out in the PubMed, Scopus, Cochrane and Embase databases, as well as a manual search. The search strategy included four keywords: “Peri-implantitis”, “Periimplantitis”, “Implant Surface Decontamination” and “Titanium Brush”. Randomized controlled studies published in the last 10 years were included and systematic reviews, in vitro studies and animal studies were excluded. Results: 142 references were found, from which only four articles met the inclusion criteria. All of the studies included in the present review reported beneficial results in terms of probing depth, gingival index and radiographic bone loss and gain after implant surface decontamination adjuvated by titanium brushes. Conclusions: titanium rotary brushes show improvements in the evolution and prognosis of peri-implant surgery, although more long-term studies are needed to draw more solid conclusions.

## 1. Introduction

At present, the use of dental implants for the rehabilitation of totally and partially edentulous patients has become the standard of treatment, showing very high success rates [1]. However, there are clinical complications described in implantology, highlighting the appearance of peri-implant disease, in which pathological changes of an inflammatory type occur in the supporting tissues that surround a loaded implant [2,3,4]. However, an effective and consensual treatment protocol has not been established [1,5], which is why different studies are being developed.

The approach to the treatment of peri-implantitis will be aimed at reducing the peri-implant pocket and eliminating the clinical signs of inflammation [6,7], since the main objectives of its treatment are to reduce the bacterial colonization of the surface of the implant, eliminate inflammation [8] and stop the progressive course of bone loss [9].

Based on the available literature, we can establish that there is a consensus that non-surgical treatment is sufficient for the treatment of mucositis, but it is not effective in solving the problem of peri-implantitis, since there have only been reported improvements in clinical parameters and it shows a clear tendency to recurrence, probably related to insufficient access to decontaminate the entire exposed surface of the implant [10,11,12]. Therefore, to treat peri-implantitis, a surgical approach is necessary in order to obtain good access for decontamination therapy, and thus be able to modify the anatomy of hard and/or soft tissues with the main objective of reducing pocket depth [10].

Prevention of peri-implant disease starts with structured and sufficient planning, including individual assessment and minimization of risk factors (smoking, compliance, oral hygiene, periodontal disease and systemic diseases), establishment of optimal hard and soft tissue conditions, choice of the correct implant design followed by a maximally atraumatic approach and regular clinical examinations with periodontal probing status. In addition, patient training sessions for optimal oral hygiene, preventive strategies such as professional cleaning of teeth and implants as well as continuous individual peri-implant examinations (probing status) to prevent peri-implant diseases should be considered [13].

Currently, there are two types of peri-implant surgical treatments: resective therapy, which consists of an apical repositioning technique with removal of soft and hard tissues to reduce the peri-implant pocket, leaving part of the implant surface exposed to facilitate the hygienic techniques of the patient, and regenerative therapy, in which we try to recover lost bone tissue through the use of biomaterials, bone grafts or substitutes and/or barrier membranes. In both techniques, we perform an access surgery, which consists of lifting a full-thickness flap to access the implant surface and, in this way, clean and decontaminate its surface and debride the bone defect [14,15].

The arrest of the progression of the disease and the bone regeneration of peri-implant defects is one of the main objectives in the surgical treatment of peri-implantitis, although there is controversy regarding its prognosis and long-term stability. The results may be affected by poor decontamination of the implant surface [16], which can be improved with the use of the recently introduced titanium brushes. These instruments are formed by a stainless-steel shaft with titanium bristles on its end, and are used by means of contra-angle handpieces of up to 900 oscillations per minute. Recent in vitro studies have shown that these brushes do not significantly alter the microsurface topography of machined nor SLA implants, and result in an increased plaque removal when compared to steel curettes [17].

Due to the lack of scientific evidence, it is considered pertinent to carry out the present systematic review on the efficacy of titanium brushes in decontamination of the implant surface in order to obtain greater reosseointegration, as well as the arrest of peri-implantitis in peri-implant surgical treatment.

Accordingly, the objectives of this systematic review were to evaluate the effectiveness of the new titanium brushes in the decontamination of the implant surface in terms of plaque index (PI), probing depth (PD), bleeding on probing (BoP) and bone loss (BL) and to evaluate the effectiveness of titanium brushes according to the type of peri-implant bone defect.

## 2. Methods

### 2.1. Protocol and Registration

This systematic review was conducted and reported in line with the “Preferred Reporting Items for Systematic reviews and Meta-Analyses” (PRISMA) guidelines [18]. The review protocol was registered in the PROSPERO database (https://www.crd.york.ac.uk/prospero/, accessed on 15 September 2019) with the registration number CRD42020163413.

### 2.2. Search Strategy and Eligibility Criteria

The search strategy was based on the PICO question: are optimal results obtained in peri-implant surgical treatment with the use of rotating titanium brushes for decontamination of the implant surface?

The inclusion criteria were randomized clinical studies on participants with peri-implantitis and no systemic disease, underlying pathology or treatment that could influence the treatment or outcome. Studies published in the last 10 years were included and no language restrictions were applied.

Systematic reviews and literature reviews, in vitro studies and animal studies were excluded.

### 2.3. Information Sources and Keywords

An advanced electronic search was performed in the Medline (via PubMed), Scopus, Cochrane and Embase databases to identify relevant studies. The search started in September 2019 and was updated in March 2021. A manual search was also carried out to identify and examine the articles that were not found in the databases and that could meet our inclusion criteria. Articles published from January 2016 to March 2021 were included.

The search strategy included 4 keywords “Peri-implantitis”, “Periimplantitis”, “Implant Surface decontamination“ and “Titanium brush”, the first two terms being MeSH. Boolean operators (“OR” and “AND”) were used to join the terms related to the research question (Table 1).

### 2.4. Study Selection

Study selection was performed independently by the same two investigators who were blinded to each other (F.G. and M.G.) on the basis of two selection phases. First, the titles and abstracts of the search results were checked. Secondly, the full texts of possibly relevant articles were checked. In addition, backward and forward tracking was performed as part of the search strategy. Backward follow-up includes selecting the reference lists of relevant articles (that is, all studies included in this systematic review, as well as other relevant systematic reviews), while forward follow-up includes searching for studies that cited the studies that were included in this systematic review. A consensus meeting between the two investigators was arranged after each step in the study selection process, where discrepancies between the selected studies were discussed. In case of doubts or disagreements between researchers, a third and fourth researcher (C.L. and E.R.) were consulted to make a final decision. The study selection process was performed with the aid of Mendeley reference management software (Elsevier, Amsterdam, The Netherlands).

#### Study Data

The variables collected in each article were: author, year and journal; type of study; sample size; implant surface decontamination method; type of surgery; follow-up time; plaque index (PI) (initial and final); probing depth (PD) (initial and final); bleeding on probing (BoP) (initial and final); bone loss (BL) (initial and final) and effects of titanium brushes in peri-implant surgery.

### 2.5. Quality Assessment (Risk of Bias)

The methodological quality of each included study was independently assessed by two assessors (F.G. and M.G.). Risk of bias was assessed using the Cochrane Collaboration [19] tool for randomized controlled trials. Furthermore, the quality of the prospective clinical study was assessed using the tool for assessing the risk of bias in nonrandomized intervention studies (ROBINS-I) [20]. A consensus meeting was held between the two reviewers to discuss possible differences in the methodological quality scores. In case of doubts or disagreements between these two reviewers, a third investigator (C.L.) was consulted to make a final decision.

## 3. Results

### 3.1. Study Selection and Flowchart

The search identified a total of 142 references related to the effects of titanium brushes on implant surface decontamination in peri-implant surgery, of which 18 were found in Pubmed, 14 in Embase, 21 in Cochrane, 88 in Scopus and 1 in the manual search based on the bibliographic references of the included articles.

After excluding 58 duplicates, the 84 remaining were assessed. Of these, 79 were excluded upon reading the title and abstract, as they did not answer our research question.

After reading the full text of the remaining five articles, one was excluded because it did not meet our inclusion criteria [21].

**Figure 1:**
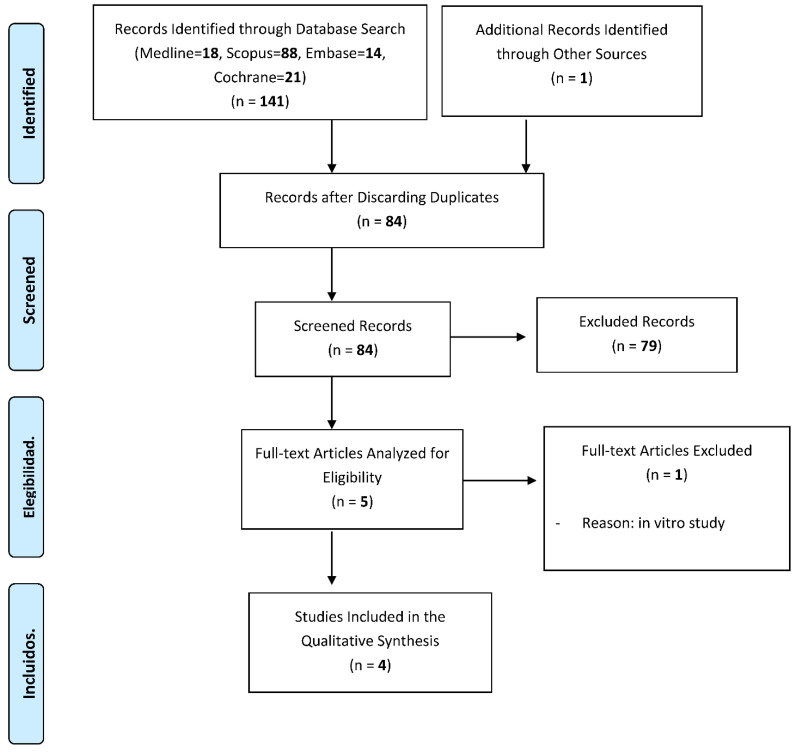
A flowchart giving a detailed overview of the study selection process.

Finally, four articles met the inclusion criteria and were included in the review [17,22,23,24]. The PRISMA flow chart (Figure 1) represents an overview of the study selection process.

### 3.2. Characteristics of the Included Studies

All the studies included in this systematic review used titanium rotary brushes as a method of decontamination of the implant surface in peri-implant resective or regenerative surgery.

The included studies reported a similar structural pattern, most of them offering a sufficient summary, a clear objective, a description of the methodology, a mention of the statistical analyses used and the relevant conclusions.

### 3.3. Qualitative Synthesis of the Included Studies

A summary of the methodology of the included studies is presented in Table 2. A summary of the quantitative parameters reported by the included studies is presented in Table 3.

The studies included in our systematic review employed a sample ranging from 24 [24] to 63 patients [23].

In the study by Tapia et al., (2018) [17] only the titanium brush was used in the experimental group as a decontamination method. However, in the other studies, an adjuvant method was also used. Two studies [22,24] also used sterile sodium chloride. In addition to these two methods to decontaminate the surface, Jepsen et al., (2016) [23] added 3% hydrogen peroxide as a chemical method. The four articles included compared this experimental group, in which a titanium brush was used, with one or two other study groups, in which ultrasonic tips, plastic curettes or abrasive air were used as an alternative method.

The predominant type of peri-implant surgery according to the studies included in our review was regenerative surgery. Only one study used resective therapy as the sole surgical treatment [22]. However, in another study [23] they performed regenerative surgery on one of the groups and resective surgery on the other. Regenerative surgery was performed with different biomaterials according to the study: biocompatible materials such as tricalcium phosphate with hydroxyapatite have been used as bone fillers [17], porous titanium granules [23,24] or xenograft [24]. These biomaterials were covered with a collagen membrane only [17], with a collagen membrane plus platelet-rich fibrin membrane in one of the groups, or with a platelet-rich fibrin membrane only in the other group [25].

The follow-up time of the patients varied between 6 months [22,24] and a year [17,23].

The variables studied were PI, mean PD, BoP and BL. With the exception of one article [17], a clear reduction in the PI was seen after the follow-up time of the patients. The mean PD, probing bleeding and BL decreased in all articles that reported these variables.

All the articles included in this review performed statistical analyses, which showed statistically significant beneficial results in terms of PD [17] (*p* = 0.007); gingival index, PD and BL [22] (*p* > 0.0001) and radiographic bone filling (bone gain) [18,19] (*p* < 0.05).

### 3.4. Risk of Bias Tool Assessment

The details of the risk of bias assessment are illustrated in Table 4 and Table 5.

Using the Cochrane Collaboration tool [19], all three randomized controlled trials were rated as good quality. The Cochrane Collaboration recommends a specific tool to assess the risk of bias in each included study. It includes a description and an assessment for each item in a “Risk of bias” table, in which each item addresses a specific aspect of the study. The rating for each item includes the answer to a question, in which the answer “Yes” indicates a low risk of bias, “No” indicates a high risk of bias and “Unclear” indicates a lack of information or uncertainty about the possible bias (Table 4).

The three randomized studies coincide in four of the seven items of the Cochrane tool, specifically in the domains of masking of the evaluation or measurement of the results; incomplete results data; selective description of the results and other sources of bias. In these four domains, all three studies are at low risk of bias.

However, they do not coincide in the first and second domains, with one study [17] presenting a low risk of bias and two of them [22,23] having an unclear risk of bias. Additionally, item three “masking of researchers, personnel involved in the study or study participants” was categorized as unclear for all of the included studies, since the treatment with or without a titanium brush cannot be masked for researchers.

The risk of bias judgments from ROBINS-I [20], including the domains before and after the intervention, is shown in Table 5. The nonrandomized study was assessed and resulted in a moderate overall risk of bias.

## 4. Discussion

The objective of this study was to present a systematic review of the available literature, analyzing the effects of the new titanium brushes for the decontamination of the implant surface, which could improve the results, evolution and prognosis of the patient’s surgical treatment of peri-implantitis.

The structured search and data extraction strategy was carried out by an individual examiner, with the limitations that this may entail. As the PRISMA statement indicates, the MEDLINE database is one of the most exhaustive sources of information in the health field, but like any database, its coverage is not complete. For this reason, we searched four databases (Medline, Scopus, Embase and Cochrane), with the aim of amplifying our search as much as possible.

The Cochrane tool [11] and ROBINS-I [20] were used to assess the risk of bias of the included studies, since they are recommended for the quality assessment of randomized controlled trials and nonrandomized studies of interventions, respectively.

The positive effect of the titanium brush has already been demonstrated during in vitro studies [25], in which it was concluded that the titanium brush appears to be more effective in the ability to remove plaque, while being softer with the implant surface than steel curettes. These results are supported by the findings of another study [26] that evaluated the effect of rotating titanium brushes in combination with four chemical agents on titanium surfaces covered by a Staphylococcus epidermis biofilm. Three different titanium surfaces were used: SLA surfaces, samples that mimic TiUnite surfaces and samples that mimic OsseoSpeed surfaces. The combination of the titanium brushes with chemical agents resulted in a greater reduction in biofilm compared to using the same chemical agents alone. These results coincide with the study by Tapia et al., (2018) [17] in which a statistically significant improvement in PD was seen in the group in which hydrogen peroxide was used as a decontamination method together with titanium brushes, compared to the control group, in which only hydrogen peroxide with ultrasound was used.

The in vitro study by Sanz-Martín et al., (2020) evaluated the decontamination efficacy of three different methods (titanium brushes, ultrasonic tip and abrasive air) on four types of implants with different depths and thread pitch. These implants were stained with a surrogate biofilm and inserted into defects designed using a 3D printer. It was observed that the effectiveness of the air abrasive was lower than that of the titanium brush or the ultrasonic tip, while there were no significant differences between the latter two. The ultrasonic tip showed a significantly higher percentage of residual staining on the implant with the highest thread pitch, while the titanium brush had higher residual staining on the implant with a pronounced reverse buttress thread design. They concluded that the thread geometry influenced the access of the decontamination devices and, in turn, their effectiveness. Implants with smaller thread pitch and thread depth values appeared to have less residual staining [27].

In the study by Ronay et al., (2017) implants were also stained for decontamination using a Gracey curette, an ultrasonic scaler and an air-powder abrasive device with a submucosal nozzle using glycine powder to evaluate the cleaning potential of commonly used implant debridement methods, simulating non-surgical peri-implantitis therapy in vitro. It was observed that the air-powder abrasive device showed significantly better results for all defect angulations. In addition, scanning electron microscopic evaluation showed considerable surface alterations after instrumentation with Gracey curettes and ultrasonic devices, whereas the glycine powder did not produce any surface alterations [28].

Regarding the effectiveness of other decontamination methods, the study by Sahrmann et al., (2015) evaluated the cleaning potential of Gracey curettes, an ultrasonic device and abrasive air for implant surface decontamination in vitro, using a bone defect simulation model. Dental implants were also stained with indelible ink and mounted on resin models. It was observed that airflow devices using glycine powders appear to be an effective therapeutic option for implant debridement in peri-implantitis defects, especially in wide defects, while producing less topographical changes on the implant surface than ultrasonic tips [29].

Additionally, following the use of abrasive air with glycine powder to decontaminate the implant surface in the study by Sahrman et al., (2013), it was concluded that although complete surface decontamination could not be achieved on any of the defects (vertical bone angulations of 90, 60, 30 and 15°), the majority of the surface could be cleaned on the larger defects [30].

With regards to titanium brushes, however, an in vitro study [31] assessed the roughness of the titanium surface and observed that the treatment with a titanium brush did not significantly change the roughness parameters, including the arithmetic mean height of the surface and the maximum height of the surface, both on machined surfaces and treated with sandblasting and acid etching.

In the study by Al-Hashedi et al., in 2016 [32], the ability of clinically available methods, such as metal and plastic curettes, titanium brushes and the Er:YAG laser to decontaminate implant surfaces was evaluated. The surface morphology, chemical composition and properties of the machined titanium discs were analyzed before and after contamination of oral biofilms by scanning electron microscopy and X-ray photoelectron spectroscopy. Biofilm contamination created an organic layer that firmly adhered to titanium surfaces. Titanium brushes were found to be more effective than curettes (metal or plastic) and the Er:YAG laser in decontaminating Ti implant surfaces, although neither technique was able to completely remove surface contamination. These results are in favor of the study by Toma et al., (2019) [22], which showed better results in the decontamination of the implant surface in the group that used titanium brushes compared to the plastic curettes group or the abrasive air group.

This effectiveness has also been demonstrated in experimental studies in animals [33]. In this study in dogs, four surface decontamination protocols were compared during the surgical treatment of peri-implantitis (group 1: Tibrush^®^ + chlorhexidine + H_2_O_2_; group 2: Tibrush^®^ + chlorhexidine; group 3: ultrasound + chlorhexidine and group 4: no treatment). The first two groups obtained similar results that showed better statistically significant results, in terms of reduction of inflammation and reduction of PD, compared to groups 3 and 4. Similarly, in the study by Tapia et al., (2018) [17] a statistically significant improvement in PD was seen in the group in which hydrogen peroxide was used as a decontamination method together with titanium brushes, compared to the control group, in which only hydrogen peroxide with ultrasound was used.

In another experimental animal study [34] carried out in six dogs in which the appearance of peri-implant disease was induced, it was concluded that mechanical decontamination of the implant surface carried out with a rotating titanium brush resulted in a marginal increase in bone level, producing a low content of inflammatory infiltrate near the marginal bone. This coincides with two studies included in our review [23,24], in which a statistically significant bone gain (*p* < 0.05) was obtained in the groups in which titanium brushes were used as an implant surface decontamination method.

## 5. Recommendations for Further Research

It is important to note that more comparative clinical studies with a larger sample and during a longer follow-up time are needed to establish firm conclusions on the subject in question.

## 6. Conclusions

All of the studies included in the present review reported beneficial results in terms of probing depth, gingival index, radiographic bone loss and gain. In addition, titanium brushes have been shown to be useful for narrow defects, because the titanium bristles can offer easier access to these spaces and can be perfectly adapted to the architecture of the implant.

Nevertheless, it should be noted that the nature of the included studies hinders the evaluation of the real added value of the titanium brush, due to their use together with other treatment modalities.

## Figures and Tables

**Table 1:** Search Strategy and results by database.

Database	Search Strategy	Results
Medline	#1 (periimplantitis) OR (peri-implantitis)	2.038
	#2 (implant surface decontamination)	172
	#3 (titanium brush)	80
	#1 AND #2 AND #3	18
Scopus	#1 (periimplantitis) OR (peri-implantitis)	8.762
	#2 (implant surface decontamination)	2.057
	#3 (titanium brush)	10.239
	#1 AND #2 AND #3	88
Cochrane	#1 (periimplantitis) OR (peri-implantitis)	21
	#2 (implant surface decontamination)	6
	#3 (titanium brush)	3
	#1 AND #2 AND #3	21
Embase	#1 (periimplantitis) OR (peri-implantitis)	3.014
	#2 (implant surface decontamination)	226
	#3 (titanium brush)	105
	#1 AND #2 AND #3	14

**Table 2:** Synthesis of the methodology and results of the studies included in the review.

Author and Year	Type of Study	Sample	Decontamination Method	Type of Surgery	Follow-Up Time	Findings
Tapia et al., (2018) [17]	RCT	**Control group**: n = 15 **Experimental group**: n = 15	**Control group**: points of US and H_2_O_2_ at 3%. **Experimental group**: = control group + titanium brushes	Regenerative	12 months	PD in the experimental group: *p* = 0.007 Rest of variables: *p* > 0.05
Toma et al., (2019) [22]	RCT	**Plastic curettes group**: n = 15 **Abrasive air group**: n = 16 **Titanium brushes group**: n = 16	**Plastic curettes group** (Gracey): with irrigation with NaCl. **Abrasive air group** (Perio-Flow^®^): glycine amino acid powder application + irrigation with NaCl. **Titanium brushes group** (Ti-Brush^®^): with irrigation with NaCl.	Resective	6 months	PI and PD in the titanium brushes group: *p* < 0.001) BL in the titanium brushes group with respect to the plastic curettes: *p* < 0.001)
Jepsen et al., (2016) [23]	RCT	**Case group**: n = 33 **Control group**: n = 30	Both groups with decontamination with titanium brushes + H_2_O_2_ al 3%.	**Case group**: regenerative	12 months	Increased bone gain in the case group: *p* < 0.0001
				**Control group**: resective.		
Guler et al., (2016) [24]	Prospective Clinical Study	**Group 1**: n = 18 **Group 2**: n = 6	**Group 1**: titanium curettes + titanium brushes **Group 2**: titanium curettes.	**Group 1**: regenerative	6 months	Increased bone gain in group 1: *p* < 0.05
				**Group 2**: regenerative		

Abbreviations (BL: bone loss; PD: probing depth; PI: plaque index; RCT: randomized clinical trial; US: ultrasounds).

**Table 3:** Synthesis of the methodology and results of the studies included in the review: quantitative parameters.

Author and Year	PI (%)		PD (mm)		BoP (%)		BL (mm)	
	Initial	Final	Initial	Final	Initial	Final	Initial	Final
Tapia et al. (2018) [17]	14.54 ± 6.12	16.56 ± 8.39	6.16 ± 1.27	3.32 ± 0.72	100 ± 0	20 ± 41	3.91 ± 0.93	1.2 ± 1.14
Toma et al. (2019) [22]	1.12 ± 0.44	0.3 ± 0.23	6.45 ± 1.87	3.98 ± 1.43	62 ± 4.7	16 ± 3.7	7.09 ± 1.23	5.88 ± 1.3
Jepsen et al. (2016) [23]	25.8 ± 36.8	21.0 ± 28.7	6.3 ± 1.3	3.5 ± 1.5	89.4 ± 20.7	33.3 ± 3.7	M: 5.55 ± 2.3 D: 5.41 ± 2.72	M: 1.98 ± 1.99 D: 1.96 ± 1.95
	24.8 ± 36.3	10.33 ± 20.0	6.3 ± 1.6	3.5 ± 1.1	85.8 ± 23.9	40.4 ± 37.1	M: 4.63 ± 4.45 D: 4.45 ± 2.23	M: 3.63 ± 2.34 D: 3.63 ± 2.32
Guler et al. (2016) [24]	0.73 ± 0.72	0.64 ± 0.52	5.28 ± 1.06	3.34 ± 0.82	50.17 ± 25.19	24.32 ± 11.22	-	BG: 1.74 ± 0.65
	0.98 ± 0.82	0.61 ± 0.66	4.72 ± 1.02	3.18 ± 0.54	63.51 ± 24.38	33.00 ± 15.51	-	BG: 1.05 ± 0.54

Abbreviations (D: distal; PI: plaque index; M: mesial; BL: bone loss; BG: bone gain; PD: probing depth; BoP: bleeding on probing).

**Table 4:** Assessing the risk of bias of randomized trials using the Cochrane tool.

Study	1	2	3	4	5	6	7	Final Assessment
Tapia et al., (2018) [17]	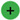	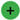	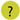	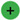	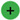	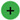	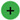	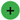
Toma et al., (2019) [22]	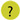	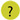	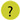	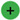	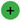	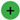	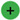	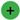
Jepsen et al., (2016) [23]	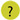	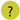	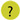	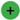	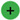	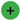	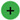	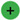

1: Generation of the randomization sequence; 2: concealment of the intervention allocation process; 3: masking of researchers, personnel involved in the study or study participants; 4: masking of the evaluation or measurement of the results; 5: incomplete results data; 6: selective description of the results; 7: other sources of bias.

**Table 5:** ROBINS-I (risk of bias judgements in nonrandomized studies of interventions).

Author and Year	Confounding	Selection of Participants	Classification of Interventions	Deviations from Intended Deviations	Missing Data	Measurement of Outcomes	Selection of Reported Results	Overall
Guler et al., (2016) [19]	Low	Low	Low	Low	Moderate	Low	Moderate	Moderate

Low: comparable to a well-performed randomized trial; moderate: sound for a nonrandomized study, but not comparable to a rigorous randomized trial; serious: presence of important problems; critical: too problematic to provide any useful evidence on the effects of intervention. Overall risk of bias: equal to the most severe level of bias found in any domain.

## Data Availability

The data presented in this study are available on request from the corresponding author.
